# Social Roles of Family and Friends Differ in Social Networks of Older Adults Who Live Alone

**DOI:** 10.1093/geroni/igab046.2371

**Published:** 2021-12-17

**Authors:** Sato Ashida, Ellen Schafer, Lena Thompson

**Affiliations:** 1 University of Iowa, Iowa City, Iowa, United States; 2 Boise State University, Boise State University, Idaho, United States; 3 University of Iowa College of Public Health, Iowa City, Iowa, United States

## Abstract

Social networks consisting of family and friends tend to better facilitate older adults’ emotional well-being than networks consisting of only family or only friends. This study assessed the heterogeneity of older adults’ network compositions based on the network members’ relationship (family vs. friends) and proximity (local vs. non-local) and evaluated the types of interactions between older adults and types of members. Adults 60 years and older living in a U.S. Midwestern city participated in a one-time structured survey (n=133), and reported about 1,730 social network members. Compared to participants who lived with others, those who lived alone reported more depressive symptoms and higher frequency of feeling lonely (p=0.002). Those who lived alone also had higher proportions of local friends in their networks than those who lived with others (p=0.02). Whereas the social roles of family and friends were similar in networks of older adults who lived with others, those who lived alone were less likely to identify family as who they co-engaged in social activities with (local family OR=0.55, non-local family OR=0.27) and who provided companionship (local family OR=0.33, non-local family OR=0.11) compared to their local friends. Having more members who co-engaged in activities was associated with lower depressive symptoms (p=0.05) and less frequency of feeling lonely (p<0.01). Providing supportive infrastructure for community-based older adults to develop and maintain co-engaging relationships with local friends may be beneficial. Network approaches can be used to identify existing network members who may be inspired to play this role.

